# Polycaprolactone Scaffolds for BONE Regeneration in Animal Models of Cranial Defects: A Systematic Review and Meta-Analysis

**DOI:** 10.3390/jfb17100495

**Published:** 2026-09-30

**Authors:** Yuehua Li, Lei Zheng, Bernadette Quah, Wei Seong Toh, Bozhi Hou, Xin Yang, Raymond Chung Wen Wong

**Affiliations:** 1 Faculty of Dentistry, National University of Singapore, National University Centre for Oral Health, 9 Lower Kent Ridge Road, Singapore 119085, Singapore; yuehuali@u.nus.edu (Y.L.);; 2 Department of Orthopaedic Surgery, Yong Loo Lin School of Medicine, National University of Singapore, Singapore 119228, Singapore

**Keywords:** Polycaprolactone, osteogenesis, preclinical, PCL, tissue engineering

## Abstract

Polycaprolactone (PCL) is a biodegradable polymer widely used in bone tissue engineering; however, its regenerative performance in cranial defect models remains incompletely characterized. This systematic review and meta-analysis evaluated the effect of PCL scaffolds on bone regeneration in preclinical cranial defect models. PubMed, Embase, Web of Science, and Scopus were systematically searched, and eligible animal studies were selected according to predefined criteria. A total of 37 studies were included in the systematic review. Data on scaffold characteristics, animal models, and osteogenic outcomes were extracted, followed by random-effects meta-analysis and meta-regression to investigate potential sources of heterogeneity. PCL-based scaffolds significantly enhanced bone regeneration at one and two months post-surgery, with pooled mean differences of 1.68 (95% CI: 0.34–3.03; *p* = 0.01) and 5.60 (95% CI: 3.48–7.72; *p* < 0.00001), respectively. The treatment effect was greatest at two months but decreased at three months and was no longer statistically significant (MD = 2.22, 95% CI: −1.36–5.80; *p* = 0.22). Extreme between-study heterogeneity was observed across the time-point analyses, limiting the consistency and generalizability of the pooled estimates. Meta-regression identified animal species as a significant moderator at one month, accounting for 28.8% of the estimated heterogeneity, whereas follow-up duration and defect size were not significant moderators. Overall, PCL-based scaffolds demonstrate a beneficial but potentially time-dependent effect on bone regeneration, while their long-term regenerative performance remains uncertain.

## 1. Introduction

Bone deficiency is a common issue in clinical treatment globally, resulting from congenital abnormalities, trauma, disease or some surgical procedures. In recent years, bone tissue engineering has emerged as a promising method to solve this problem, with biodegradable PCL-based scaffolds attracting considerable attention for their favourable properties. Polycaprolactone (PCL) is one kind of biodegradable polymer and has low cytotoxicity, but it also has some limitations, including poor cell adhesion due to its hydrophobicity and suboptimal degradation rates. To overcome these challenges, it is commonly used in combination with other components such as β-tricalcium phosphate(β-TCP), hyaluronic acid and bioactive peptides, and these biocomponents benefit it with better tissue integration [1,2,3].

The current clinical application of PCL scaffolds for cranial osteogenesis is encouraging with respect to safety and early signs of osteogenesis. In 2023, three patients underwent PCL mesh treatment to promote osteogenesis, suggesting that PCL-based polymers are beginning to transition into clinical trial studies [4]. However, it is limited by small sample sizes, heterogeneous scaffold compositions and manufacturing methods, variable outcome measures (radiologic vs. histologic), and relatively short follow-up durations. These limitations hinder quantitative pooling of efficacy outcomes and underscore the need for standardized radiologic/histologic endpoints and longer prospective studies [5].

Despite the increasing interest in PCL scaffolds for bone tissue engineering, their osteogenic performance in animal models of cranial defects has not been systematically evaluated across different follow-up periods. The available studies vary in animal species, defect characteristics, scaffold architecture, and fabrication or surface-modification approaches, which may contribute to differences in reported bone-regeneration outcomes. In addition, variations in outcome reporting may limit direct comparisons and quantitative synthesis across studies. Therefore, a systematic evaluation of the available preclinical evidence is needed to characterize the observed effects of PCL scaffolds in cranial defect models and to explore potential sources of variation. In particular, synthesizing bone volume fraction (BV/TV) data from eligible PCL scaffold groups and their corresponding empty-defect controls at different follow-up time points may provide a structured assessment of the available evidence while highlighting the limitations associated with between-study heterogeneity.

Therefore, our systematic review and meta-analysis aimed to evaluate bone regeneration following implantation of PCL scaffolds in animal models of cranial defects by comparing BV/TV outcomes between eligible PCL scaffold groups and their corresponding empty-defect controls at different follow-up time points. We also aimed to explore potential sources of heterogeneity in the reported treatment effects. This review will give valuable clues into the current state of PCL scaffold development and provide some insights for clinical application and future research directions.

## 2. Methodology

### 2.1. Objective

This systematic review evaluated the osteogenic potential of PCL degradable scaffolds via analysis of their effect in cranial defect animal model.

### 2.2. Review Protocol

The registered systematic review (PROSPERO number: CRD420261435691) collected studies until 3 July 2026 from PubMed, Embase, Web of Science, and Scopus databases. The search process adhered to the guidelines outlined by PRISMA. The PICO framework guided the selection process—Population: Animal models of cranial defects. Intervention: Eligible PCL scaffolds. An eligible PCL scaffold was defined as a scaffold in which PCL was the sole scaffold material, without the incorporation of additional materials or bioactive agents into the scaffold matrix. Comparison: Empty defect. Outcome: Bone regeneration, with BV/TV as the quantitative outcome used for meta-analysis.

### 2.3. Search Strategy

A comprehensive literature search was conducted in PubMed, Embase, Web of Science, and Scopus databases. The search strategy combined terms related to PCL, osteogenesis and bone regeneration, in vivo or animal studies, and cranial defects. Database-specific search terms and Boolean operators were adapted as appropriate for each database. The complete search strategies for all four databases are provided in Supplementary search strategies. Two reviewers (Yuehua Li and Raymond Chung Wen Wong) conducted an independent search, examining the titles and abstracts of studies in line with the following inclusion and exclusion criteria to identify potentially relevant works for this review. Subsequently, three reviewers (Yuehua Li, Zheng Lei and Bernadette Quah) assessed the selected studies and discussed their eligibility. The selected studies then underwent full-text screening and further analysis; any studies that failed to meet the eligibility criteria were excluded during this process.

### 2.4. Eligibility Criteria

#### 2.4.1. Inclusion Criteria

Original articles exploring cranial regeneration of scaffolds based on PCL, either alone or combined with other elements, were selected in this review. The inclusion standards were:

• Article written in English;
• Scaffolds based on PCL for bone regeneration;
• In vivo cranial defect model used in the research;
• Accessible information on osteogenic data.

#### 2.4.2. Exclusion Criteria

Erratum letters, letters to the editor, case reports, reviews, and other non-original articles were excluded. Additionally, studies without an empty defect and eligible PCL groups BV/TV (%) data were also excluded after eligibility analysis.

### 2.5. Data Extraction

Following the identification of all included studies, the information recorded is as below: author, publication year, animal strain/sex/age/body weight/defect size/defect number/follow-up time. PCL fabrication method, scaffold size, pore size, porosity percentage, structure feature, scaffold modification; BV/TV (%) mean ± SD and acquisition time/repeat number. For BV/TV data extraction, numerical values reported in the text or tables were extracted directly. When data were presented only graphically, the corresponding values were extracted from the figures using WebPlotDigitizer (Automeris, v5). Data extraction was performed by Yuehua Li. Before extracting data from each figure, the axes were calibrated according to the corresponding axis scales. The extracted values were recorded and organized for quantitative synthesis. The handling of dispersion measures was based on the information available in each study.

### 2.6. Assessment of Quality and Risk of Bias

The SYRCLE risk of bias tool [6] was applied to evaluate the risk of bias in all studies included, with a summary of the assessment presented in Table 1. This tool is adapted from the Cochrane RoB tool to address specific types of bias relevant to animal intervention studies. Each domain was classified as low risk, high risk, or unclear risk according to the information reported in the original study. Low risk was assigned when sufficient information supported that the relevant methodological criterion had been adequately addressed. High risk was assigned when the reported information indicated a methodological concern likely to introduce bias. Unclear risk was assigned when the available information was insufficient to determine whether the criterion had been adequately addressed. For summary purposes, the overall risk-of-bias category was derived using the following operational rules: studies with at least one high-risk domain were classified as overall high risk; studies with no high-risk domains but at least two unclear domains were classified as overall moderate risk; studies with all domains rated as low risk or only one unclear risk were classified as overall low risk.

### 2.7. Meta-Analysis and Meta-Regression

Meta-analysis was performed using RevMan (Review Manager). Continuous outcomes (BV/TV, %) were summarized as mean differences (MDs) with 95% confidence intervals (CIs), using a random-effects model. Statistical heterogeneity was assessed using the Chi^2^ test and quantified by the I^2^ statistic. Subgroup analyses were performed according to follow-up time, with assessments at 1 month, 1–2 months, and >2 months classified as early, middle, and late stages respectively. For the pig model, a previously published study reported incomplete bone repair at 12 weeks, with predominantly soft tissue observed histologically [7]. Therefore, the study with a 5-week follow-up was classified as an early-stage assessment, considering the relatively slower bone-healing process in pigs.

Meta-regression analyses were subsequently performed at time points with substantial between-study heterogeneity to explore potential sources of variability using R studio (version 4.5.2). Follow-up duration and defect size were evaluated as continuous moderators, whereas animal species was treated as a categorical moderator. Random-effects meta-regression models were fitted using restricted maximum likelihood (REML), and moderator effects were assessed using the omnibus QM test. The proportion of between-study heterogeneity accounted for by each moderator was evaluated using R^2^. *p* < 0.05 was considered statistically significant.

**Table 1:** Risk of bias assessment of the included animal studies using the SYRCLE tool.

Author, Year	Selection Bias			Performance Bias		Detection Bias				Attrition Bias	Reporting Bias	Other	Overall RoB
	Sequence Generation	Baseline Characteristics	Allocation Concealment	Random Housing	Blinding	Random Outcome Assessment	Gross Blinding	Imaging Blinding	Histology Blinding	Incomplete Outcome Data	Selective Outcome Reporting	Other Sources of Bias	
A. T. Qureshi, 2015 [8]	Unclear	Low	Unclear	Unclear	Unclear	Unclear	Unclear	Unclear	Unclear	Low	Low	Low	Moderate
M. Rezai-Rad, 2015 [9]	Low	Moderate	Low	Low	Unclear	Unclear	Unclear	Unclear	Unclear	Low	Low	Low	Moderate
K. K. Moncal, 2018 [10]	Unclear	Moderate	Unclear	Unclear	Unclear	Unclear	Unclear	Unclear	Unclear	Low	Low	Low	Moderate
T. Dai, 2022 [11]	Low	Moderate	Low	Low	Unclear	Unclear	Unclear	Unclear	Unclear	Low	Low	Low	Moderate
X. Wang, 2024 [12]	Unclear	Low	Unclear	Unclear	Unclear	Unclear	Unclear	Unclear	Unclear	Low	Low	Low	Moderate
K. Wang, 2024 [13]	Unclear	Low	Unclear	Unclear	Unclear	Unclear	Unclear	Unclear	Unclear	Low	Low	Low	Moderate
B. S. Kim, 2014 [14]	Low	Moderate	Low	Low	Unclear	Unclear	Unclear	Unclear	Unclear	Low	Low	Low	Moderate
X. Li, 2019 [15]	Low	Moderate	Low	Low	Unclear	Unclear	Unclear	Unclear	Unclear	Low	Low	Low	Moderate
W. Wang, 2019 [16]	Unclear	Low	Unclear	Low	Unclear	Unclear	Unclear	Unclear	Unclear	Low	Low	Low	Moderate
Y. Huan, 2023 [17]	Low	Low	Low	Unclear	Unclear	Unclear	Unclear	Unclear	Unclear	Low	Low	Low	Moderate
L. Zhong, 2020 [18]	Low	Low	Unclear	Unclear	Unclear	Unclear	Unclear	Unclear	Unclear	Low	Low	Low	Moderate
S. S. R. Bojedla, 2023 [19]	Low	Low	Low	Low	Unclear	Unclear	Unclear	Unclear	Unclear	Low	Low	Low	Moderate
Y. Deng, 2019 [20]	Unclear	Low	Unclear	Unclear	Unclear	Unclear	Unclear	Unclear	Unclear	Low	Low	Low	Moderate
K. Jiang, 2024 [21]	Low	Low	Unclear	Unclear	Unclear	Unclear	Unclear	Unclear	Unclear	Low	Low	Low	Moderate
T. Dai, 2024 [22]	Unclear	Low	Unclear	Unclear	Unclear	Unclear	Unclear	Unclear	Unclear	Low	Low	Low	Moderate
J. Wu, 2021 [23]	Unclear	Low	Unclear	Unclear	Unclear	Unclear	Unclear	Unclear	Unclear	Low	Low	Low	Moderate
Ö. Toprak, 2021 [24]	Unclear	Low	Unclear	Unclear	Unclear	Unclear	Unclear	Unclear	Unclear	Low	Low	Low	Moderate
A. Padalhin, 2021 [25]	Unclear	Low	Unclear	Unclear	Unclear	Unclear	Unclear	Unclear	Unclear	Low	Low	Low	Moderate
K. Khanna, 2017 [26]	Unclear	Low	Unclear	Unclear	Unclear	Unclear	Unclear	Unclear	Unclear	Low	Low	Low	Moderate
M. He, 2024 [27]	Unclear	Low	Unclear	Unclear	Unclear	Unclear	Unclear	Unclear	Unclear	Low	Low	Low	Moderate
J. Jensen, 2016 [28]	Low	Low	Low	Low	Unclear	Unclear	Unclear	Unclear	Unclear	Low	Low	Low	Moderate
N. Lv, 2025 [29]	Low	Low	Unclear	Unclear	Unclear	Unclear	Unclear	Unclear	Unclear	Low	Low	Low	Moderate
S. He, 2023 [30]	Low	Low	Unclear	Unclear	Unclear	Unclear	Unclear	Unclear	Unclear	Low	Low	Low	Moderate
M. Janmohammadi, 2024 [31]	Unclear	Low	Unclear	Unclear	Unclear	Unclear	Unclear	Unclear	Unclear	Low	Low	Low	Moderate
H. Tang, 2026 [32]	Low	Low	Unclear	Unclear	Unclear	Unclear	Unclear	Unclear	Unclear	Low	Low	Low	Moderate
H. Zhao, 2025 [33]	Low	Low	Unclear	Unclear	Unclear	Unclear	Unclear	Unclear	Unclear	Low	Low	Low	Moderate
S. Liu, 2026 [34]	Unclear	Low	Unclear	Unclear	Unclear	Unclear	Unclear	Unclear	Unclear	Low	Low	Low	Moderate
X. Liu, 2025 [35]	Low	Low	Unclear	Unclear	Unclear	Unclear	Unclear	Unclear	Unclear	Low	Low	Low	Moderate
X. Xu, 2023 [36]	Unclear	Low	Unclear	Unclear	Unclear	Unclear	Unclear	Unclear	Unclear	Low	Low	Low	Moderate
F. Xing, 2022 [37]	Low	Low	Unclear	Unclear	Unclear	Unclear	Unclear	Unclear	Unclear	Low	Low	Low	Moderate
Q. Wang, 2020 [38]	Low	Low	Unclear	Unclear	Unclear	Unclear	Unclear	Unclear	Unclear	Low	Low	Low	Moderate
I. J. de Souza Araújo, 2022 [39]	Low	Low	Unclear	Unclear	Unclear	Unclear	Unclear	Unclear	Unclear	Low	Low	Low	Moderate
Y. Guo, 2025 [40]	Low	Low	Unclear	Unclear	Unclear	Unclear	Unclear	Unclear	Unclear	Low	Low	Low	Moderate
H. Xu, 2022 [41]	Low	Low	Unclear	Unclear	Unclear	Unclear	Unclear	Unclear	Unclear	Low	Low	Low	Moderate
J. Y. Kim, 2018 [42]	Low	Low	Unclear	Unclear	Unclear	Unclear	Unclear	Unclear	Unclear	Low	Low	Low	Moderate
X. Li, 2025 [43]	Low	Low	Unclear	Unclear	Unclear	Unclear	Unclear	Unclear	Unclear	Low	Low	Low	Moderate
B. Wang, 2023 [44]	Low	Low	Unclear	Unclear	Unclear	Unclear	Unclear	Unclear	Unclear	Low	Low	Low	Moderate

## 3. Results

**Figure 1:**
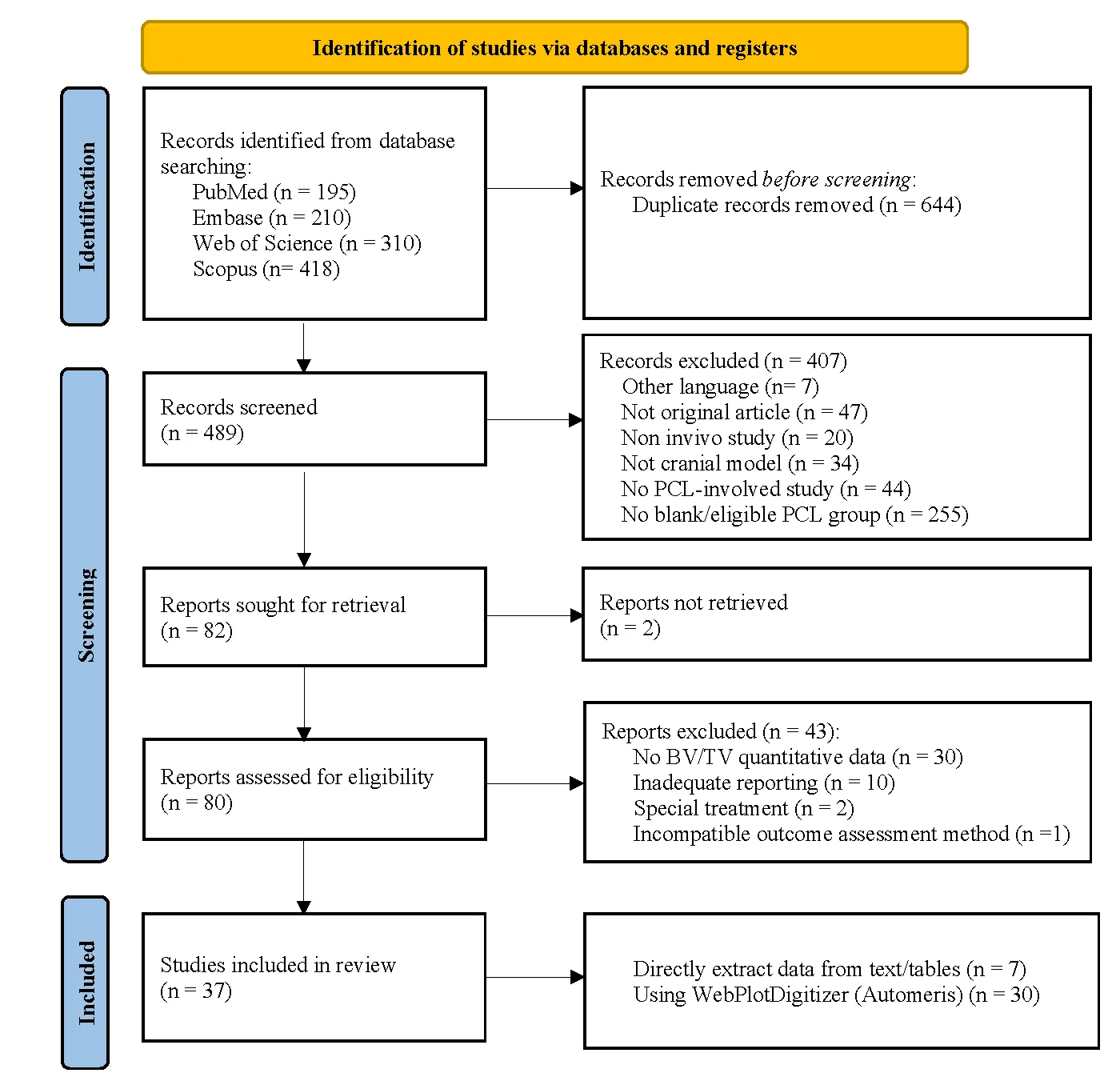
PRISMA flow diagram of search strategy and study selection process.

The flowchart in Figure 1 illustrates the search strategy employed in this study. Initially, 1133 articles were retrieved from the above-mentioned four databases. After removing duplicates, 489 abstracts were screened for eligibility. Following a second round of qualitative review, 82 full-text articles were further evaluated, with 2 studies not retrieved. Of the 80 reports assessed for eligibility, 43 were excluded for the following reasons: lack of quantitative BV/TV data (n = 30), inadequate reporting (n = 10), special treatment (n = 2), and incompatible outcome assessment methods (n = 1). Ultimately, 37 studies were included for further analysis.

The specifics of each study are presented in Table 2, Table 3 and Table 4. Table 2 provides animal details on their strain, sex, age, weight, defect size, defect number and follow-up time. Table 3 outlines details related to PCL used, including PCL fabrication method, scaffold size, pore size, porosity, structural features and surface modification. BV/TV values, along with their corresponding acquisition times and repeat numbers, are presented in Table 4.

**Figure 2:**
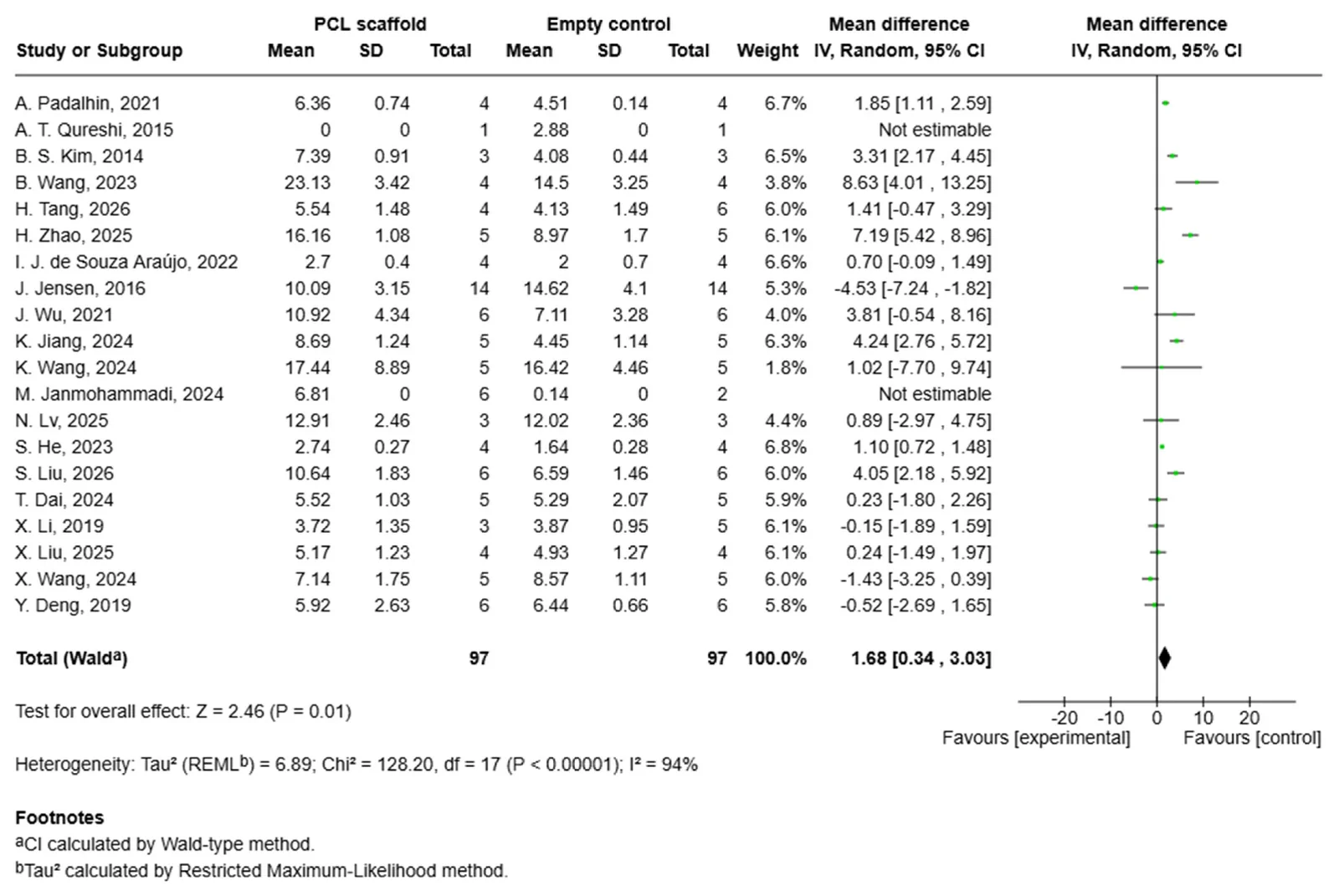
One-month outcomes of PCL scaffolds in animal cranial defect models [8,12,13,14,15,20,21,22,23,25,28,29,30,31,32,33,34,35,39,44].

During the first month after surgery, data from 20 studies were available for analysis, with 97 animals in both the PCL and empty-defect groups (Figure 2). Two studies were not estimable because the reported data did not allow calculation of the mean difference. The pooled analysis demonstrated a significant beneficial effect of PCL scaffolds on bone regeneration, yielding a mean difference of 1.68 (95% CI: 0.34–3.03). This effect reached statistical significance (Z = 2.46, *p* = 0.01). However, considerable between-study heterogeneity was identified (Chi^2^ = 128.20, df = 17, *p* < 0.00001; I^2^ = 94%), suggesting substantial variability in the magnitude of the treatment effect across studies. Therefore, the pooled mean difference should be interpreted as an average effect across the included studies rather than evidence of a consistent effect in every animal model or experimental setting.

**Figure 3:**
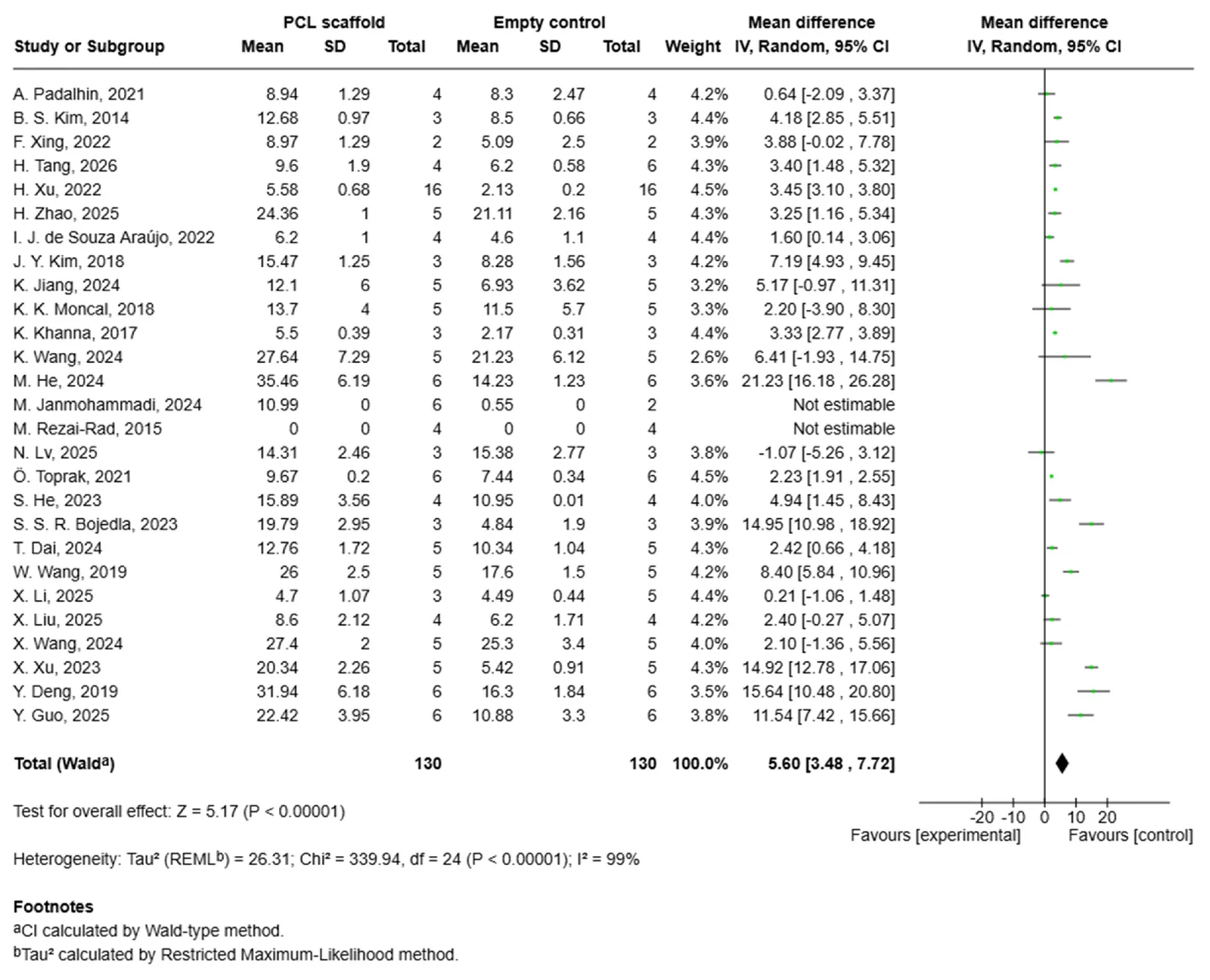
Osteogenic analysis of PCL scaffolds in animals during second month [9,10,12,13,14,16,19,20,21,22,24,25,26,27,29,30,31,32,33,35,36,37,39,40,41,42,43].

For the second month’s data, 27 studies were identified, involving 130 animals in each treatment group (Figure 3). A total of 25 studies provided estimable data for quantitative synthesis while the remaining two studies were excluded because the reported data were insufficient to calculate the mean difference. Overall, PCL scaffold treatment was associated with a significantly greater extent of bone regeneration than empty defects, with a pooled mean difference of 5.60 (95% CI: 3.48–7.72). The overall treatment effect was highly significant (Z = 5.17, *p* < 0.00001). Notably, the results were accompanied by very high heterogeneity (Chi^2^ = 339.94, df = 24, *p* < 0.00001; I^2^ = 99%), indicating that the treatment effect varied markedly among the included studies. Accordingly, the pooled estimate represents an average effect and should not be interpreted as demonstrating a uniform response across the included experimental models.

**Figure 4:**
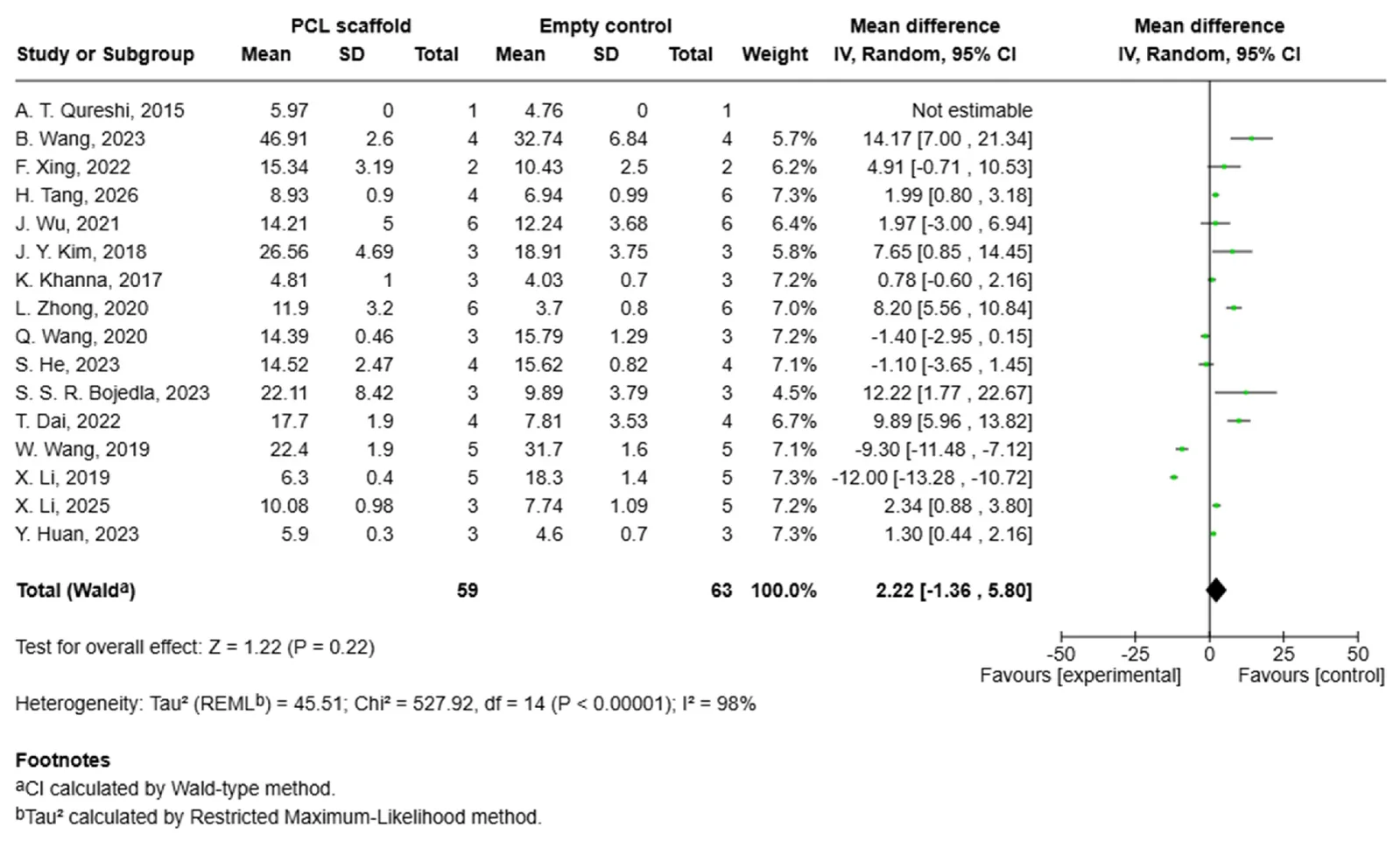
Meta-analysis of osteogenesis after 3 months in cranial defect model [8,11,15,16,17,18,19,23,26,30,32,37,38,42,43,44].

After two months, 16 studies contributed data, comprising 59 animals in the PCL scaffold group and 63 in the empty-defect group (Figure 4). And one study was not included for quantitative synthesis because the reported data were insufficient to calculate the mean difference. The pooled estimate supported PCL scaffolds, with a mean difference of 2.22, although the corresponding 95% CI (−1.36 to 5.80) crossed the null value. Accordingly, the overall effect did not reach statistical significance (Z = 1.22, *p* = 0.22). Marked heterogeneity persisted at this time point (Chi^2^ = 527.92, df = 14, *p* < 0.00001; I^2^ = 98%), further indicating substantial differences in treatment effects across studies. Together with the confidence interval crossing the null value, this finding suggests that the available evidence does not establish a consistent or statistically significant effect at this later follow-up stage.

## 4. Meta Regression Analysis

Meta-regression analyses were performed to explore whether follow-up duration, defect size, and animal species contributed to the observed between-study heterogeneity. For animal species, rats were prespecified as the reference category because rat models were the most frequently represented animal model among the included studies.

During the first month post-surgery, follow-up duration and defect size were not significant moderators of the treatment effect. In contrast, animal species significantly moderated the treatment effect (QM = 9.51, *p* = 0.023), accounting for 28.8% of the estimated between-study heterogeneity. The treatment effect was significantly lower in mouse models than in the reference animal category (*p* = 0.035), whereas the other species-specific contrasts were not statistically significant.

At two and three months post-surgery, none of the evaluated moderators significantly explained the observed between-study heterogeneity (all *p* > 0.05; Table 5A,B).

## 5. Discussion

Recent research has investigated PCL with both natural and synthetic materials to balance mechanical strength and biological function. The combination of PCL with various materials presents a promising strategy for designing scaffolds with properties tailored to specific tissue engineering needs [45].

This systematic review and meta-analysis examined the effects of PCL scaffolds on bone regeneration in animal cranial defect models. PCL scaffolds provide early and middle osteogenic benefits, with pooled mean differences of 1.68 (95% CI: 0.34–3.03) and 5.60 (95% CI: 3.48–7.72), respectively. Surface modifications and composite designs have been shown to accelerate osteogenic differentiation and improve bone-forming potential in vitro and in vivo [46].

Although statistically significant pooled effects were observed at the early and intermediate follow-up stages, these findings should be interpreted in the context of the substantial between-study heterogeneity. The high I^2^ values at one month (94%), one to two months (99%), and beyond two months (98%) indicate that the observed treatment effects varied considerably across the included studies. Therefore, statistical significance of the pooled estimate should not be equated with consistency of the effect. The pooled mean differences are best interpreted as average effects across the included studies, with the magnitude and reproducibility of the effect in individual models remaining uncertain.

Several methodological and biological differences may have contributed to the observed heterogeneity, including animal species, defect size and configuration, scaffold architecture, fabrication methods, surface modifications, follow-up duration, and differences in outcome acquisition and reporting. The meta-regression identified animal species as a statistically significant moderator at one month, accounting for 28.8% of the estimated heterogeneity; however, this finding does not fully explain the variability observed across studies. At later follow-up time points, the moderators examined were not statistically significant. Other study-level characteristics may also contribute to the observed variation, but their effects could not be reliably assessed in the present analysis. These potential explanations should therefore be regarded as hypotheses rather than established determinants of treatment response.

However, the magnitude of the observed benefit decreased at three months and was no longer statistically significant (MD = 2.22, 95% CI: −1.36–5.80). This finding indicated that the available evidence was insufficient to determine whether the regenerative benefits of PCL scaffolds persist over longer periods. The attenuation of the treatment effect at the later time point is consistent with the possibility that scaffold degradation characteristics may influence bone regeneration; however, this relationship cannot be established from the present data. PCL is known for its relatively slow degradation, which can allow the scaffold to maintain structural support for an extended period. One possible hypothesis is that prolonged scaffold persistence could influence the space available for bone deposition and tissue replacement. However, this hypothesis could not be directly evaluated in the present meta-analysis because degradation kinetics were not consistently reported across the included studies. However, this interpretation should be considered cautiously, as scaffold degradation kinetics were not consistently reported across the included studies and could not be directly evaluated in the present meta-analysis.

Several limitations should be considered when interpreting these findings. Substantial heterogeneity persisted even after meta-regression, indicating that important sources of variability could not be fully assessed. Although random-effects models were used and potential moderators were explored, considerable variability remained unexplained. In addition, the pooled estimates may be sensitive to individual studies, particularly where study populations, defect configurations, or scaffold characteristics differ substantially. Differences in scaffold design, PCL properties, fabrication techniques, composite components, defect configuration, and outcome assessment methods may have contributed to the observed variability, but these factors were not sufficiently reported across studies to permit comprehensive moderator analyses. These original descriptions of these features were retained in Table 3 to accurately represent the source publications. The pooled estimates should be interpreted as the observed effects of eligible PCL scaffold groups compared with empty defects, rather than as definitive estimates applicable to every PCL formulation or surface treatment. In addition, the animal-species findings represent study-level associations and should not be interpreted as evidence of a causal effect of animal species on scaffold performance. Although the follow-up categories used in this review represent approximate analytical intervals and were explored as a continuous moderator, the available study-level data and the exploratory nature of the meta-regression limit the extent to which species-specific healing trajectories can be distinguished. Consequently, differences between the time-based pooled estimates should be interpreted cautiously.

Overall, the present findings support the potential of PCL scaffolds to promote bone regeneration, particularly during the early and intermediate stages of healing, with the greatest pooled treatment effect observed at two months. However, the attenuation and loss of statistical significance at three months, together with the substantial residual heterogeneity, indicate that the long-term regenerative performance of PCL remains uncertain. Future studies should prioritize standardized animal models and defect designs, consistent reporting of scaffold properties and degradation, longer follow-up periods, and the development of composite or modified PCL scaffolds with improved biological activity and controlled degradation to enhance the durability and translational relevance of PCL-based bone regeneration.

## 6. Conclusions

This meta-analysis demonstrated that PCL scaffolds significantly promoted bone regeneration in cranial defect animal models at one and two months post-surgery, with pooled mean differences of 1.68 (95% CI: 0.34–3.03) and 5.60 (95% CI: 3.48–7.72), respectively. The treatment effect was most pronounced at two months, whereas the magnitude of the beneficial effect decreased by three months and was no longer statistically significant (MD = 2.22, 95% CI: −1.36–5.80, *p* = 0.22). However, substantial heterogeneity was observed at all follow-up stages (I^2^ = 94%, 99%, and 98%, respectively), indicating considerable variability in treatment effects across studies. Meta-regression identified animal species as a statistically significant moderator of the treatment effect at one month ((QM = 9.51), *p* = 0.023), with the model attributing 28.8% of the estimated between-study heterogeneity to this moderator. The treatment effect was significantly lower in mouse models than in the reference animal category (*p* = 0.035), whereas the other species-specific contrasts were not statistically significant. At later follow-up time points, no statistically significant moderators were identified among follow-up duration, defect size, and animal species. Overall, the findings suggest a potentially beneficial average effect of PCL scaffolds during early and intermediate healing stages; however, the substantial heterogeneity limits conclusions regarding the consistency and generalizability of this effect. The long-term regenerative performance of PCL scaffolds remains uncertain.

## Figures and Tables

**Table 2:** Summary of characteristics of animal model.

Paper ID	Animal Model	Strain	Sex	Age	Body Weight	Defect Size	Defect Number	Follow-Up Time
A. T. Qureshi, 2015 [8]	mice	CD-1 nude mice	Male	42 day	20–40 g	4 mm	1	4, 12 weeks
M. Rezai-Rad, 2015 [9]	rat	Sprague Dawley	Male	5 month	NR	5 mm	2	4, 8 weeks
K. K. Moncal, 2018 [10]	rat	Fischer 344 white	Male	8 week	NR	5 mm	2	8 weeks
T. Dai, 2022 [11]	rat	Wistar	Male	Adult	220–250 g	5 mm	2	4, 8, 16 weeks
X. Wang, 2024 [12]	rat	Sprague Dawley	Male	8 week	200–250 g	5 mm	2	1, 2 months
K. Wang, 2024 [13]	rat	Sprague Dawley	Female	8 week	300 g	5 mm	2	4, 8 weeks
B. S. Kim, 2014 [14]	rabbit	New Zealand White	Male	3–4 month	NR	8 mm	4	2, 8 weeks
X. Li, 2019 [15]	rabbit	New Zealand White	NR	NR	2.2–3.2 kg	8 mm	2	6, 12 weeks
W. Wang, 2019 [16]	rat	Wistar	Male	NR	300–350 g	5 mm × 5 mm	1	60 days
Y. Huan, 2023 [17]	dog	Beagle dogs	Male	18–20 month	15–17 kg	2 cm	1	9 months
L. Zhong, 2020 [18]	rabbit	New Zealand White	NR	12 weeks	2.5 ± 0.2 kg	10 mm	2	12 weeks
S. S. R. Bojedla, 2023 [19]	rabbit	New Zealand White	Male	NR	1.9–2.1 kg	8 mm	4	6, 12 weeks
Y. Deng, 2019 [20]	mice	CD-1 mice	NR	6–8 weeks	NR	4 mm	2	4, 8 weeks
K. Jiang, 2024 [21]	rat	Sprague Dawley	Female	10 weeks	300 g	5 mm	2	4, 8 weeks
T. Dai, 2024 [22]	rat	Sprague Dawley	Male	NR	200 ± 10 g	5 mm	2	4, 8 weeks
J. Wu, 2021 [23]	rat	Sprague Dawley	Female	Adult	220 ± 20 g	5 mm	2	4, 12 weeks
Ö. Toprak, 2021 [24]	rat	Wistar	Female	NR	250–350 g	5 mm	1	8 weeks
A. Padalhin, 2021 [25]	rat	Sprague Dawley	NR	NR	250–300 g	8 mm	1	4, 8 weeks
K. Khanna, 2017 [26]	rat	Wistar	Male	6 month	NR	8 mm	1	6, 12 weeks
M. He, 2024 [27]	rat	Sprague Dawley	Male	4 weeks	100–120 g	5 mm	2	8 weeks
J. Jensen, 2016 [28]	Pig	Danish Landrace	Female	537 ± 19 days	190 ± 32 kg	15 mm diamater; 10 mm thickness	6	5 weeks
N. Lv, 2025 [29]	rat	Sprague Dawley	Female	NR	NR	5 mm	1	4, 8 weeks
S. He, 2023 [30]	rat	Sprague Dawley	Male	7 weeks	NR	5 mm	2	1 week, 2 months, 4 months
M. Janmohammadi, 2024 [31]	rat	Wistar	Female	4 weeks	200–250 g	8 mm	1	4, 8 weeks
H. Tang, 2026 [32]	rat	Sprague Dawley	Male	8 weeks	NR	5 mm	2	1, 2, 3 months
H. Zhao, 2025 [33]	rat	Sprague Dawley	Male	NR	200–250 g	5 mm	2	4, 8 weeks
S. Liu, 2026 [34]	rat	Sprague Dawley	Female	12 weeks	NR	5 mm	2	30 days
X. Liu, 2025 [35]	rat	Sprague Dawley	Male	8 weeks	200–250 g	5 mm	2	4, 8 weeks
X. Xu, 2023 [36]	rat	NR	NR	4 weeks	NR	6 mm	1	8 weeks
F. Xing, 2022 [37]	rabbit	New Zealand White	NR	Adult	2.5–3.0 kg	8 mm	2	6, 12 weeks
Q. Wang, 2020 [38]	rat	Sprague Dawley	Male	NR	NR	5 mm	2	12 weeks
I. J. de Souza Araújo, 2022 [39]	rat	Fischer 344	Male	6 weeks	200–220 g	5 mm	1	4, 8 weeks
Y. Guo, 2025 [40]	rat	Sprague Dawley	Male	NR	180–200 g	5 mm	2	8 weeks
H. Xu, 2022 [41]	rabbit	New Zealand White	Female	NR	3 kg on average	6 mm	4	8 weeks
J. Y. Kim, 2018 [42]	rabbit	New Zealand White	Male	12 weeks	3.0–3.5 kg	8 mm	4	6, 12 weeks
X. Li, 2025 [43]	rat	Sprague Dawley	Male	8 weeks	NR	5 mm	2	4, 8, 12 weeks
B. Wang, 2023 [44]	rat	Sprague Dawley	Male	6 weeks	190–230 g	5 mm	2	4, 12 weeks

NR: Not reported.

**Table 3:** Fabrication data of the PCL-based scaffolds.

Paper ID	Fabrication Method	Scaffold Size	Pore Size (μm)	Porosity (%)	Structural Features	Surface Modification
A. T. Qureshi, 2015 [8]	NR	4 mm diameter; 1 mm thickness	NR	NR	NR	NR
M. Rezai-Rad, 2015 [9]	NR	5 mm × 0.5 mm	200–300	NR	Porous	NR
K. K. Moncal, 2018 [10]	3D printing	NR	212.83 ± 25.56	43.33 ± 6.46	NR	NR
T. Dai, 2022 [11]	Electrospin	5 mm diameter, 3 mm height	NR	73.6	Porous	NR
X. Wang, 2024 [12]	Electrospin	thickness: 500 ± 50 μm	NR	NR	Janus Structure Membrane	NR
K. Wang, 2024 [13]	Electrospin	5 mm diameter	NR	NR	Nano-topological structure	NR
B. S. Kim, 2014 [14]	Leaching	NR	200–300	85.5 ± 0.7	Teflon molds	NR
X. Li, 2019 [15]	Moulding/ salt leaching	NR	180 ± 10	86.0	Porous	NR
W. Wang, 2019 [16]	Extrusion-based additive manufacturing	5 mm × 5 mm	680	NR	Porous	NR
Y. Huan, 2023 [17]	Extrusion-based 3D printing	20 × 20 × 2.1 mm	NR	NR	NR	NR
L. Zhong, 2020 [18]	Extrusion-based 3D printing	10 mm diameter, 3 mm height	442 ± 40	68.52 ± 5.89	Porous	NR
S. S. R. Bojedla, 2023 [19]	Extrusion-based 3D printing	7.5 × 1.1 mm^3^	NR	NR	Porous	NR
Y. Deng, 2019 [20]	Electrospin	Cover the defects	NR	NR	Fibrous membranes	NR
K. Jiang, 2024 [21]	Electrospin	Fill the defects	NR	NR	Nanofibrous scaffold (NS) with/without nanotopology (TNS)	NR
T. Dai, 2024 [22]	3D printing	Fill the defects	500	NR	Porous	5 M NaOH for 15 min
J. Wu, 2021 [23]	Electrospin	5 mm diameter	NR	NR	Three-layered membranes with wafers	NR
Ö. Toprak, 2021 [24]	Electrospin	NR	NR	NR	Fibrous membrane	NR
A. Padalhin, 2021 [25]	Electrospin	8 mm diameter	NR	NR	Porous membrane	NR
K. Khanna, 2017 [26]	Electrospin	NR	3.07 ± 1.08	NR	Porous	NR
M. He, 2024 [27]	Electrostatic spinning	NR	NR	NR	NR	NR
J. Jensen, 2016 [28]	Extrusion-based 3D printing	NR	NR	NR	NR	5 M NaOH for 3 h
N. Lv, 2025 [29]	Electrospin	Cover the defects	NR	NR	Porous membrane	NR
S. He, 2023 [30]	Electrospin	5 mm diameter	NR	NR	Porous	NR
M. Janmohammadi, 2024 [31]	Extrusion-based 3D printing	8 mm diameter	NR	NR	Porous scaffold	1 M sodium hydroxide for 6 h
H. Tang, 2026 [32]	Extrusion-based 3D printing	5 mm diameter, 1 mm height	NR	80.52 ± 0.78	Porous scaffold	NR
H. Zhao, 2025 [33]	Electrospin	NR	NR	NR	NR	NR
S. Liu, 2026 [34]	Thermal molding	5 mm diameter	NR	NR	Flake-shape porous structure	NR
X. Liu, 2025 [35]	Extrusion-based 3D printing	Well-fitted to the cranial defect sites	NR	NR	Porous scaffolds	20 M NaOH solution for 3 h
X. Xu, 2023 [36]	Electrospin	NR	NR	NR	Nanofibrous membrane	NR
F. Xing, 2022 [37]	Extrusion-based 3D printing	NR	BPS: 476.19 ± 97.74 TPS: 466.17 ± 82.7	BPS: 50.50 ± 1.13 TPS: 50.34 ± 0.91	BPS: bare 3D-printed PCL porous scaffold TPS: nanotopological porous structure	Immersed in an acetic acid and distilled water (volume ratio at 77%/23%) mixed solution for 5 min
Q. Wang, 2020 [38]	Extrusion-based 3D printing	5 mm diameter, 1.5 mm height	NR	NR	Porous scaffolds	Immersed in a 0.02 g/mL 1,6-hexanediamine/isopropanol solution for 20 min
I. J. de Souza Araújo, 2022 [39]	Electrospin	16 × 16 mm^2^; cover the defects	NR	NR	Fibrous porous scaffold	NR
Y. Guo, 2025 [40]	Extrusion-based 3D printing	NR	NR	NR	Membrane	NR
H. Xu, 2022 [41]	3D printing	6 mm diameter, 2.1 mm height	NR	35	Inhomogeneous porous scaffold	NR
J. Y. Kim, 2018 [42]	Extrusion-based 3D printing	8 mm diameter, 2 mm height	300	NR	Porous scaffolds	PBS for 1 h to improve wetting.
X. Li, 2025 [43]	Extrusion-based 3D printing	5 mm diameter, 1 mm height	NR	67.97 ± 1.89	Porous scaffold	NR
B. Wang, 2023 [44]	melt electrowritten printing	5 mm diameter, 2 mm height	NR	NR	Porous scaffold	Simulated body fluid for 7 d

NR: Not reported.

**Table 4:** Overview of BV/TV data, acquisition time, and repetition counts.

Analysis Month	Paper ID	Time Point	Animal Model	BV/TV_Mean (%) ± SD	Number
Early stage ≤1 month	B. S. Kim, 2014 [14]	2 weeks	rabbit	Empty defect: 4.08 ± 0.44	3
				PCL: 7.39 ± 0.91	
	A. T. Qureshi, 2015 [8]	4 weeks	mice	Empty defect: 2.88	4
				PCL: 0	
	X. Wang, 2024 [12]	4 weeks	rat	Empty defect: 8.57 ± 1.11	5
				PCL: 7.14 ± 1.75	
	K. Wang, 2024 [13]	4 weeks	rat	Empty defect: 16.42 ± 4.64	5
				PCL: 17.44 ± 8.89	
	Y. Deng, 2019 [20]	4 weeks	mice	Empty defect: 16.3 ± 1.84	6
				PCL: 31.94 ± 6.18	
	K. Jiang, 2024 [21]	4 weeks	rat	Empty defect: 4.45 ± 1.14	5
				PCL: 8.69 ± 1.24	
	T. Dai, 2024 [22]	4 weeks	rat	Empty defect: 5.29 ± 2.07	5
				PCL: 5.52 ± 1.03	
	J. Wu, 2021 [23]	4 weeks	rat	Empty defect: 7.11 ± 3.28	6
				PCL: 10.92 ± 4.34	
	A. Padalhin, 2021 [25]	4 weeks	rat	Empty defect: 4.51 ± 0.14	4
				PCL: 6.36 ± 0.74	
	J. Jensen, 2016 [28]	5 weeks	pig	Empty defect: 14.62 ± 4.10	14
				PCL: 10.09 ± 3.15	
	N. Lv, 2025 [29]	4 weeks	rat	Empty defect: 12.02 ± 2.36	3
				PCL: 12.91 ± 2.46	
	S. He, 2023 [30]	1 week	rat	Empty defect: 1.64 ± 0.28	4
				PCL: 2.74 ± 0.27	
	M. Janmohammadi, 2024 [31]	4 weeks	rat	Empty defect: 0.14	Empty defect: 2 PCL: 6
				PCL: 6.81	
	H. Tang, 2026 [32]	1 month	rat	Empty defect: 4.13 ± 1.49	Empty defect: 6 PCL: 4
				PCL: 5.54 ± 1.48	
	H. Zhao, 2025 [33]	4 weeks	rat	Empty defect: 8.97 ± 1.70	5
				PCL: 16.16 ± 1.08	
	S. Liu, 2026 [34]	30 days	rat	Empty defect: 6.59 ± 1.46	6
				PCL: 10.64 ± 1.83	
	X. Liu, 2025 [35]	4 weeks	rat	Empty defect: 4.93 ± 1.27	4
				PCL: 5.17 ± 1.23	
	I. J. de Souza Araújo, 2022 [39]	4 weeks	rat	Empty defect: 2.0 ± 0.7	4
				PCL: 2.7 ± 0.4	
	X. Li, 2025 [43]	4 weeks	rat	Empty defect: 3.87 ± 0.95	Empty defect: 5 PCL: 3
				PCL: 3.72 ± 1.35	
	B. Wang, 2023 [44]	4 weeks	rat	Empty defect: 14.50 ± 3.25	4
				PCL: 23.13 ± 3.42	
Middle stage >1–2 months	M. Rezai-Rad, 2015 [9]	8 weeks	rat	Empty defect: 0.00 ± 0.00	4
				PCL: 0.00 ± 0.00	
	K. K. Moncal, 2018 [10]	8 weeks	rat	Empty defect: 11.5 ± 5.7	5
				PCL: 13.7 ± 4	
	X. Wang, 2024 [12]	2 months	rat	Empty defect: 25.3 ± 3.4	5
				PCL: 27.4 ± 2.0	
	K. Wang, 2024 [13]	8 weeks	rat	Empty defect: 21.23 ± 6.12	5
				PCL: 27.64 ± 7.29	
	B. S. Kim, 2014 [14]	8 weeks	rabbit	Empty defect: 8.50 ± 0.66	3
				PCL: 12.68 ± 0.97	
	W. Wang, 2019 [16]	60 days	rat	Empty defect: 17.6 ± 1.5	5
				PCL: 26 ± 2.5	
	S. S. R. Bojedla, 2023 [19]	6 weeks	rabbit	Empty defect: 4.84 ± 1.9	3
				PCL: 19.79 ± 2.95	
	Y. Deng, 2019 [20]	8 weeks	mice	Empty defect: 6.44 ± 0.66	6
				PCL: 5.92 ± 2.63	
	K. Jiang, 2024 [21]	8 weeks	rat	Empty defect: 6.93 ± 3.62	5
				NS PCL: 12.10 ± 6.00	
	T. Dai, 2024 [22]	8 weeks	rat	Empty defect: 10.34 ± 1.04	5
				PCL: 12.76 ± 1.72	
	Ö. Toprak, 2021 [24]	8 weeks	rat	Blank: 7.44 ± 0.34	6
				PCL: 9.67 ± 0.20	
	A. Padalhin, 2021 [25]	8 weeks	rat	Empty defect: 8.30 ± 2.47	4
				PCL: 8.94 ± 1.29	
	K. Khanna, 2017 [26]	6 weeks	rat	Empty defect: 2.17 ± 0.31	3
				PCL: 5.50 ± 0.39	
	M. He, 2024 [27]	8 weeks	rat	Empty defect: 14.23 ± 1.23	6
				PCL: 35.46 ± 6.19	
	N. Lv, 2025 [29]	8 weeks	rat	Empty defect: 15.38 ± 2.77	3
				PCL: 14.31 ± 2.46	
	S. He, 2023 [30]	2 months	rat	Empty defect: 10.95 ± 0.01	4
				PCL: 15.89 ± 3.56	
	M. Janmohammadi, 2024 [31]	8 weeks	rat	Empty defect: 0.55	Empty defect: 2 PCL group: 6
				PCL: 10.99	
	H. Tang, 2026 [32]	2 months	rat	Empty defect: 6.20 ± 0.58	Empty defect: 6 PCL: 4
				PCL: 9.60 ± 1.90	
	H. Zhao, 2025 [33]	8 weeks	rat	Empty defect: 21.11 ± 2.16	5
				PCL: 24.36 ± 1.00	
	X. Liu, 2025 [35]	8 weeks	rat	Empty defect: 6.20 ± 1.71	4
				PCL: 8.60 ± 2.12	
	X. Xu, 2023 [36]	8 weeks	rat	Empty defect: 5.42 ± 0.91	5
				PCL: 20.34 ± 2.26	
	F. Xing, 2022 [37]	6 weeks	rabbit	Empty defect: 5.09 ± 2.5	2
				PCL: 8.97 ± 1.29	
	I. J. de Souza Araújo, 2022 [39]	8 weeks	rat	Empty defect: 4.6 ± 1.1	4
				PCL: 6.2 ± 1.0	
	Y. Guo, 2025 [40]	8 weeks	rat	Empty defect: 10.88 ± 3.3	6
				PCL: 22.42 ± 3.95	
	H. Xu, 2022 [41]	8 weeks	rabbit	Empty defect: 2.13 ± 0.20	16
				PCL: 5.58 ± 0.68	
	J. Y. Kim, 2018 [42]	6 weeks	rabbit	Empty defect: 8.28 ± 1.56	3
				PCL: 15.47 ± 1.25	
	X. Li, 2025 [43]	8 weeks	rat	Empty defect: 4.49 ± 0.44	Empty defect: 5 PCL: 3
				PCL: 4.70 ± 1.07	
Late stage >2 months	A. T. Qureshi, 2015 [8]	12 weeks	mice	Empty defect: 4.76	1
				PCL: 5.97	
	T. Dai, 2022 [11]	16 weeks	rat	Empty defect: 7.81 ± 3.53	4
				PCL: 17.7 ± 1.9	
	X. Li, 2019 [15]	12 weeks	rabbit	Empty defect: 18.3 ± 1.4	5
				PCL: 6.3% ± 0.4	
	W. Wang, 2019 [16]	120 days	rat	Empty defect: 31.7 ± 1.6	5
				PCL: 22.4 ± 1.9	
	Y. Huan, 2023 [17]	9 months	dog	Empty defect: 4.6 ± 0. 7	3
				PCL: 5.9 ± 0.3	
	L. Zhong, 2020 [18]	12 weeks	rabbit	Empty defect: 3.7 ± 0.8	6
				PCL: 11.9 ± 3.2	
	S. S. R. Bojedla, 2023 [19]	12 weeks	rabbit	Empty defect: 9.89 ± 3.79	3
				PCL: 22.11 ± 8.42	
	J. Wu, 2021 [23]	12 weeks	rat	Empty defect: 12.24 ± 3.68	6
				PCL: 14.21 ± 5.00	
	K. Khanna, 2017 [26]	12 weeks	rat	Empty defect: 4.03 ± 0.70	3
				PCL: 4.81 ± 1.00	
	S. He, 2023 [30]	4 months	rat	Empty defect: 15.62 ± 0.82	4
				PCL: 14.52 ± 2.47	
	H. Tang, 2026 [32]	3 months	rat	Empty defect: 6.94 ± 0.99	Empty defect: 6 PCL: 4
				PCL: 8.93 ± 0.90	
	F. Xing, 2022 [37]	12 weeks	rabbit	Empty defect: 10.43 ± 2.50	2
				BPS: 15.34 ± 3.19	
	Q. Wang, 2020 [38]	12 weeks	rat	Empty defect: 15.79 ± 1.29	3
				PCL: 14.39 ± 0.46	
	J. Y. Kim, 2018 [42]	12 weeks	rabbit	Empty defect: 18.91 ± 3.75	3
				PCL: 26.56 ± 4.69	
	X. Li, 2025 [43]	12 weeks	rat	Empty defect: 7.74 ± 1.09	Empty defect: 5 PCL: 3
				PCL: 10.08 ± 0.98	
	B. Wang, 2023 [44]	12 weeks	rat	Empty defect: 32.74 ± 6.84	4
				PCL: 46.91 ± 2.60	

The “Number” column indicates the number of defects contributing to the BV/TV data in each group, not necessarily the number of independent animals. A single sample-size value is shown when the PCL scaffold and empty-defect groups had equal sample sizes; separate values are provided when they differed.

**Table 5:** (**A**) Overall tests of moderators in meta-regression analyses. (**B**) Species-specific coefficients from meta-regression analyses.

(**A**)						
**Postoperative Time**	**Moderator**	**k**	**QM**	**df**	***p*** **-Value**	**R^2^ (%)**
1 month	Follow-up weeks	20	0.334	1	0.563	0
	Defect size	20	0.703	1	0.402	0
	Animal species	20	9.509	3	0.023	28.8
1–2 months	Follow-up weeks	27	0.019	1	0.891	0
	Defect size	27	0.148	1	0.701	0
	Animal species	27	3.742	2	0.154	6.4
After 2nd month	Follow-up weeks	16	0.099	1	0.753	0
	Defect size	16	0.01	1	0.922	0
	Animal species	16	0.163	3	0.983	0
(**B**)						
**Postoperative Time**	**Animal Species**	**β Coefficient**	**SE**	**Z**	***p*** **-Value**	**95% CI**
1 month	Mice vs. rat	−4.258	2.019	−2.109	0.035	−8.216 to −0.300
	Pig vs. rat	−6.987	3	−2.329	0.02	−12.867 to −1.108
	Rabbit vs. rat	0.853	2.726	0.313	0.754	−4.490 to 6.195
1–2 months	Mice vs. rat	10.748	5.742	1.872	0.061	−0.505 to 22.001
	Rabbit vs. rat	1.677	2.575	0.651	0.515	−3.370 to 6.723
After 2nd month	Mice vs. rat	−0.646	7.767	−0.083	0.934	−15.869 to 14.577
	Rabbit vs. rat	1.527	4.345	0.352	0.725	−6.989 to 10.044
	Dog vs. rat	−0.556	7.779	−0.072	0.943	−15.803 to 14.691

Note: β coefficients represent the difference in treatment effect between each species and the reference category (rat), with positive values indicating a greater estimated treatment effect and negative values indicating a lower estimated treatment effect relative to rat models.

## Data Availability

All data and details of the analysis that are not included in the manuscript will be made available upon reasonable request.

## Supplementary Materials

The following supporting information can be downloaded at: https://www.mdpi.com/article/10.3390/jfb17100495/s1.
