# Supplementary material for: Polycaprolactone Scaffolds for BONE Regeneration in Animal Models of Cranial Defects: A Systematic Review and Meta-Analysis
Source: J Funct Biomater. 2026 Sep 30;17(10):495. doi: 10.3390/jfb17100495 (PMC13706555; doi:10.3390/jfb17100495)
Supplement: Supplementary file 1 [file jfb-17-00495-s001.zip › jfb-4416988-supplementary.pdf]

Complete search strategies for all databases

The complete database-specific search strategies used to identify potentially relevant studies are provided below.

S1. PubMed

#1 "polycaprolactone"[Title/Abstract] OR "poly-caprolactone"[Title/Abstract] OR "polycaprolacton*"[Title/Abstract] OR "poly-epsilon-caprolactone"[Title/Abstract] OR "poly-epsilon-caprolactone"[Title/Abstract] OR "poly-epsilon-caprolactone"[Title/Abstract] OR "poly-e-caprolactone"[Title/Abstract] OR "caprolactone polymer"[Title/Abstract] OR ("caprolactone"[Title/Abstract] AND "polymer*"[Title/Abstract]) OR ("2-oxepanone"[Title/Abstract] AND "homopolymer*"[Title/Abstract]) OR "epsilon-caprolactone"[Title/Abstract]

#2 "osteogen*"[Title/Abstract] OR "ossificatio*"[Title/Abstract] OR "bone formation"[Title/Abstract] OR "bone develop*"[Title/Abstract] OR "bone mineralization"[Title/Abstract] OR "skelet* formation"[Title/Abstract] OR "skelet* develop*"[Title/Abstract] OR "skeletogen*"[Title/Abstract] OR "Bone Regeneration"[Title/Abstract] OR "bone repair"[Title/Abstract] OR "bone healing"[Title/Abstract] OR "osteoinduction"[Title/Abstract] OR "osteoconduction"[Title/Abstract] OR "osteoproduction"[Title/Abstract] OR "osteoblast*"[Title/Abstract] OR "osteoprogenitor*"[Title/Abstract] OR "Osteogenesis"[MeSH Terms] OR "Bone Development"[MeSH Terms] OR "Bone Regeneration"[MeSH Terms]

#3 "in vivo"[Title/Abstract] OR "invivo"[Title/Abstract] OR "animal experiment*"[All Fields] OR "animal study"[All Fields] OR "animal studies"[All Fields] OR "preclinical"[All Fields] OR "clinical trial"[All Fields] OR "clinical studies"[All Fields]

#4 "skul*"[All Fields] OR "craniu*"[All Fields] OR "calvari*"[All Fields] OR "cranial defect*"[All Fields] OR "craniofacial defect*"[All Fields]

#1 AND #2 AND #3 AND #4

S2. Embase

('polycaprolactone'/exp OR 'poly caprolactone':ti,ab OR polycaprolactone*:ti,ab OR 'poly(caprolactone*)':ti,ab OR 'poly(epsilon-caprolactone)':ti,ab OR 'poly(ε-caprolactone)':ti,ab OR 'caprolactone polymer':ti,ab OR ('caprolactone':ti,ab AND polymer*:ti,ab) OR ('pcl':ti,ab AND (polymer*:ti,ab OR biomaterial*:ti,ab OR scaffold*:ti,ab))) AND ('osteogenesis'/exp OR 'osteogenesis' OR osteogen*:ti,ab OR ossificatio*:ti,ab OR 'bone formation':ti,ab OR 'bone development':ti,ab OR 'bone mineralization':ti,ab OR 'skeletal formation':ti,ab OR 'skeletal development':ti,ab OR skeletogen*:ti,ab OR 'bone regeneration':ti,ab OR 'bone repair':ti,ab OR 'bone healing':ti,ab OR osteoinduction:ti,ab OR osteoconduction:ti,ab OR osteoproduction:ti,ab) AND ('clinical study'/exp OR 'clinical study' OR 'clinical trial'/exp OR 'clinical trial' OR preclinical*:ti,ab OR 'animal experiment'/exp OR 'animal experiment' OR in) AND vivo:ti,ab AND (skul*:ti,ab OR craniu*:ti,ab OR calvari*:ti,ab OR cranial defect*:ti,ab OR 'craniofacial defect*':ti,ab)

S3. Web of Science

TS = (("polycaprolactone*" OR "poly-caprolactone" OR "poly(caprolactone*)" OR "poly(epsilon-caprolactone)" OR "poly(ε-caprolactone)" OR "caprolactone polymer" OR ("caprolactone" AND polymer*) OR ("PCL" AND (polymer* OR biomaterial* OR scaffold*)))) AND TS=(osteogen* OR ossificatio* OR "bone formation" OR "bone develop*" OR "bone mineralization" OR "skelet* formation" OR "skelet* develop*" OR skeletogen* OR "bone regeneration" OR "bone repair" OR "bone healing" OR osteoinduction OR osteoconduction OR osteoprotection) AND TS = ("in vivo" OR preclinical* OR "animal experiment*" OR "animal study" OR "animal studies" OR "clinical study" OR "clinical trial" OR human*) AND ALL=(skul* OR craniu* OR calvari* OR "cranial defect*" OR "craniofacial defect*")

S4. Scopus

#1 TITLE-ABS-KEY(polycaprolactone* OR "poly-caprolactone" OR "poly(caprolactone)" OR "poly(epsilon-caprolactone)" OR "poly(epsilon caprolactone)" OR "caprolactone polymer" OR (caprolactone AND polymer*) OR (PCL AND (polymer* OR biomaterial* OR scaffold*))) AND TITLE-ABS-KEY(osteogen* OR ossificatio* OR "bone formation" OR "bone develop*" OR "bone mineralization" OR "skelet* formation" OR "skelet* develop*" OR skeletogen* OR "bone regeneration" OR "bone repair" OR "bone healing" OR osteoinduction OR osteoconduction OR osteoproduction) AND TITLE-ABS-KEY("in vivo" OR preclinical* OR "animal experiment*" OR "animal study" OR "animal studies" OR "clinical study" OR "clinical trial" OR human*)

#2 TITLE-ABS-KEY (skul* OR craniu* OR calvari* OR "cranial defect*" OR "craniofacial defect*")

#1 AND #2
